# Five-Year Incidence of Chronic Kidney Disease (Stage 3-5) and Associated Risk Factors in a Spanish Cohort: The MADIABETES Study

**DOI:** 10.1371/journal.pone.0122030

**Published:** 2015-04-09

**Authors:** Miguel A. Salinero-Fort, Francisco J. San Andrés-Rebollo, Carmen de Burgos-Lunar, Paloma Gómez-Campelo, Rosa M. Chico-Moraleja, Ana López de Andrés, Rodrigo Jiménez-García, MADIABETES Group

**Affiliations:** 1 Gerencia de Atención Primaria. Gerencia Adjunta de Planificación y Calidad, Servicio Madrileño de Salud. Instituto de Investigación Hospital Universitario La Paz (IdiPAZ), Red de Investigación en Servicios de Salud en Enfermedades Crónicas (REDISSEC), Madrid, Spain; 2 Centro de Salud Las Calesas, Servicio Madrileño de Salud, Madrid, Spain; 3 Servicio de Medicina Preventiva, Hospital Universitario La Paz, Servicio Madrileño de Salud. Instituto de Investigación Hospital Universitario La Paz (IdiPAZ), Red de Investigación en Servicios de Salud en Enfermedades Crónicas (REDISSEC), Madrid, Spain; 4 Plataforma de Apoyo al Investigador Novel, Instituto de Investigación Hospital Universitario La Paz (IdiPAZ), Madrid, Spain; 5 Unidad de Medicina Interna, Hospital Central de la Defensa Gómez Ulla, Servicio Madrileño de Salud, Madrid, Spain; 6 Preventive Medicine and Public Health Department, Rey Juan Carlos University, Madrid, Spain; University of Glasgow, UNITED KINGDOM

## Abstract

**Objective:**

To evaluate the incidence rate of Chronic Kidney Disease (CKD) stage 3-5 (persistent decreased kidney function under 60 mL/min per 1.73 m^2^) among patients with type 2 diabetes over five years, to identify the risk factors associated with CKD, and develop a risk table to predict five-year CKD stage 3-5 risk stratification for clinical use.

**Design:**

The MADIABETES Study is a prospective cohort study of 3,443 outpatients with type 2 diabetes mellitus, sampled from 56 primary health care centers (131 general practitioners) in Madrid (Spain).

**Results:**

The cumulative incidence of CKD stage 3-5 at five-years was 10.23% (95% CI = 9.12–11.44) and the incidence density was 2.07 (95% CI = 1.83–2.33) cases per 1,000 patient-months or 2.48 (95% CI = 2.19–2.79) cases per 100 patient-years. The highest hazard ratio (HR) for developing CKD stage 3-5 was albuminuria ≥300 mg/g (HR = 4.57; 95% CI= 2.46-8.48). Furthermore, other variables with a high HR were age over 74 years (HR = 3.20; 95% CI = 2.13–4.81), a history of Hypertension (HR = 2.02; 95% CI = 1.42–2.89), Myocardial Infarction (HR= 1.72; 95% IC= 1.25–2.37), Dyslipidemia (HR = 1.68; 95% CI 1.30–2.17), duration of diabetes mellitus ≥ 10 years (HR = 1.46; 95% CI = 1.14-1.88) and Systolic Blood Pressure >149 mmHg (HR = 1.52; 95% CI = 1.02–2.24).

**Conclusions:**

After a five-year follow-up, the cumulative incidence of CKD is concordant with rates described in Spain and other countries. Albuminuria ≥ 300 mg/g and age over 74 years were the risk factors more strongly associated with developing CKD (Stage 3-5). Blood Pressure, lipid and albuminuria control could reduce CKD incidence of CKD in patients with T2DM.

## Introduction

Diabetic nephropathy develops in approximately 40% of all type 2 diabetes mellitus (T2DM) patients and is characterized by persistent albuminuria, elevated blood pressure (BP) and a progressive decline in kidney function leading toward end-stage renal disease. In addition, these patients have a high risk of cardiovascular disease, which further increases with deteriorating renal function [1].

The definition of Chronic Kidney Disease (CKD) is based on the presence of kidney damage (proteinuria, haematuria, or anatomical abnormality) or decreased kidney function for three months or more, irrespective of clinical diagnosis. CKD stages 3–5 are defined by a glomerular filtration rate (GFR) <60 mL/min per 1.73 m^2^, according to the KDOQI classification [2]. The estimated GFR (eGFR) has proven to be a significant, independent risk factor for cardiovascular morbidity and mortality in patients with T2DM [3, 4].

However, to our knowledge, there are not enough studies analyzing the incidence of sustained impaired eGFR and its association with risk factors in Southern Europe [5,6].

We conducted a population based cohort study of Spanish people with T2DM to assess the incidence of CKD stage 3–5 (persistent decreased kidney function under 60 mL/min per 1.73 m^2^) over five years, to identify the risk factors associated with the development of this disease, and to construct a risk table to predict five-year CKD stage 3–5 risk stratification for clinical use.

## Materials and Methods

### Study Population and Design

The Madrid Diabetes Study (MADIABETES Study) is a prospective cohort study of 3,443 T2DM outpatients which has been described in detail elsewhere [7]. Briefly, the subjects were sampled from 56 primary health care centers in the metropolitan area of Madrid (Spain). Study participants were selected by simple random sampling by participating general practitioners (n = 131), using the list of patients with a T2DM diagnosis in their computerized clinical records. This method of sample selection, based on the computerized clinical records, has been previously validated for epidemiological studies in our setting [8]. The gold standard definition of T2DM used in our study comes from the diagnostic criteria developed by the American Diabetes Association and found in their Consensus Statement for diabetic subjects [9], and that was still in effect in 2007.

Data were collected by general practitioners at baseline visit (2007) and annually during the follow-up period (2008–2012). These data were recorded in electronic Case Report Forms. Last observation carried forward was used to impute missing anthropometric values for patients with incomplete data during the follow-up period.

**Fig 1:**
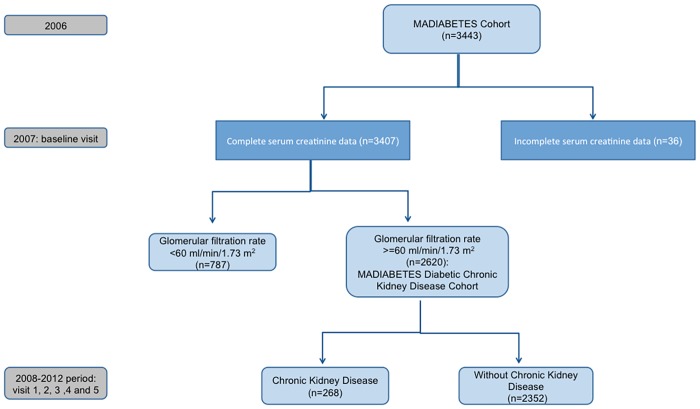
Flow diagram of participants.

Inclusion criteria were: age ≥30 years and a previous diagnosis of T2DM. Exclusion criteria were: Type 1 Diabetes Mellitus, homebound patients, presence of CKD at baseline, defined as a baseline GFR under 60 mL/min/1.73m^2^ (n = 787), and incomplete serum creatinine data (n = 36). Of the 2,620 individuals followed-up until 2012, 249 died in the period 2007–2012. The flow diagram of participants is shown in Fig 1.

After being recorded, all data were internally audited to ensure quality. This involved the random selection of 50 participating general practitioners and the review of the clinical records they produced. Strong data consistency was found (higher than 88% for all variables).

Our outcome variable was renal dysfunction incidence (CKD stage 3–5 K/DOQI) defined as estimated GFR< 60 mL/min/1.73 m^2^ at any visit, and an average successive estimated GFR of less than 60 mL/min/1.73 m^2^, among individuals free of CKD at baseline. The Modification of Diet in Renal Disease formula for estimated GFR has been shown to be an accurate indicator for CKD status [10].

To calculate incidence density the observation period for each patient was defined as the number of months from the date of their first visit in 2008 until one of the following options occurred: 1) the date CKD stage 3–5 K/DOQI was identified, 2) the date of the last visit registered, 3) date of death or, 4) 31 December 2012 (last visit).

Death data were taken from General Practitioners records. The death certificate database provided by the National Institute of Statistics was used to identify those lost to follow up due to death before the end point of the study (31 December 2012).

The median follow-up period for patients was 60 months (Interquartile range [IQR] = 24).

### Clinical Examination and Biochemistry

All patients underwent anamnesis, physical examination, and biochemical tests. The following variables were collected at baseline visit: age, gender and duration of T2DM (years). Further data was collected at baseline and each follow-up visit (at least once per year in routine clinical practice conditions): fasting plasma glucose (FPG), glycated haemoglobin (HbA1c), systolic (SBP) and diastolic blood pressure (DBP), total cholesterol, triglycerides (TG), high-density lipoprotein cholesterol (HDL-C), low-density lipoprotein cholesterol (LDL-C), albuminuria, smoking status (current smoker, former smoker, non-smoker), use of hypoglycemic and cardiovascular medications (antihypertensive agents, statins, aspirin), body mass index (BMI), history of cardiovascular events (myocardial infarction or stroke) and hypertension.

BMI was calculated as weight/height^2^ (Kg/m^2^), and patients with a BMI >30 were considered obese. Blood Pressure (BP) was measured twice using a checked, calibrated sphygmomanometer. After a 5 minute rest period, the first reading was taken, followed by a second reading 5 minutes later. History of hypertension was defined as SBP >140 mmHg and/or DBP >90 mmHg at baseline or use of antihypertensive drugs.

Dyslipidemia was defined when any of the following lipid alterations were present: total cholesterol >250 mg/dl or triglycerides >200 mg/dl; LDL-c >130 mg/dl or HDL-c <35/45 mg/dl (male/female), and metabolic syndrome was defined according to the modified criteria of the NCEP-ATP III [11].

Persistent albuminuria was defined as a urinary albumin excretion rate >30 mg/g in at least two of three consecutive samples. HbA1c was measured using high-performance liquid chromatography (Diabetes Control and Complications Trial [DCCT]-aligned) [12].

Cardiovascular risk was calculated following the REGICOR formula (a calibration of the Framingham algorithm adapted for Spain) for each patient [13].

Patients with a cardiovascular risk of 10% over ten years were considered moderate or high risk [14].

### Sample size

The sample size was estimated using the Poisson distribution and taking into account the following assumptions: 5% alpha risk, 20% beta risk and 10% loss rate. Therefore, 30 patients with albuminuria>300 mg/g and 1,800 patients with albuminuria <300 mg/g are necessary to detect a relative risk of 3.5 and a 10% of incidence of CKD in the group of not exposed patients.

### Statistical Analysis

Descriptive data were expressed as mean and standard deviation and median and IQR. Comparison of continuous variables between two groups was performed using the Student’s t-test for data that were normally distributed, the Mann–Whitney U test for non-normal distributions, and the chi-square test for categorical variables.

The cumulative incidence of CKD was calculated by taking the number of new cases as the numerator, and the total initial population excluding deaths and the number of CKD cases at baseline as the denominator. Patient-years at risk of developing CKD were calculated from the baseline appointment date to either the date of death, date of CKD event, loss to follow-up or the end of the study period. Incidence density was calculated as the number of new cases divided by patient-years at risk. The cumulative incidence and incidence density of CKD were calculated for the entire sample, and stratified by duration of T2DM, and confidence intervals were compared to analyze incidence rates of differences according to duration of T2DM.

A multivariable extension of Cox proportional hazard analysis was used to estimate the adjusted hazard ratios (HR) and corresponding 95% Confidence Intervals (CI) of independently associated predictors of developing incident CKD (stage 3–5 K/DOQI). Initially, a multivariable model was constructed by applying backwards elimination to a set of candidate predictors chosen as potential risk and confounding factors according to previous studies [15,16]. The following data were analyzed: age, gender, diabetes duration, BMI, BP, albuminuria, dyslipidemia, HbA1c, use of hypoglycemic drugs (insulin, oral antidiabetic agents) and cardiovascular medications (antihypertensive agents, statins, aspirin), cardiovascular disease (hypertension, myocardial infarction [AMI], ischemic stroke, congestive heart failure, peripheral vascular disease) and diabetic microvascular complications (retinopathy, neuropathy). With the exception of age, gender, diabetes duration and history of hypertension, all predictors were included in the model as time-varying covariate, that is to say measured repeatedly across time. Only those covariates with p<0.05 were withheld in the model, except for gender, and all possible suspected interactions between independent variables were assessed. In the final model baseline SBP was also included but as a fixed variable. The proportional hazards assumptions were tested examining the covariates*time interaction. In each case, the interaction term was not significant, thus supporting the proportional hazards assumptions.

The predictive accuracy of the multivariable Cox model was evaluated using the Harrell’s C index, which is equivalent to the area under the receiver operating characteristic curve for binary dependent variables replacing time-varying covariate by average value. Results of Harrell’s C index range from 0.5 (no discrimination for predicting CKD) to 1.0 (perfect discrimination) [17]. However, the C-index must be interpreted with caution as it is not often constructed with time-dependent variables.

Finally, regression-based coefficients were used to develop a CKD predicting risk table [18]. Predictor variables were included if they were statistically significant without establishing a hierarchy between them. All possible suspected interactions between independent variables were assessed. None of these interactions yielded a significant association. A lower CKD probability (2.8%) was calculated in the absence of risk variables, and a higher one was calculated in the presence of all of them (96.7%). Between these extremes, a total of 288 combinations of CKD probabilities were obtained.

The analysis of the results with hierarchical or multilevel models was unnecessary, as there is no evidence of the variance between the primary health care centers for the HbA1c variable being different from zero (p = 0.34); in addition, the Coefficient Correlation Intraclass has a value of 0.036.

All calculations were performed using SPSS v.21.0 software for Windows and STATA v11.1SE. Significance was set at p value <0.05 for differences with a probable type I error.

### Ethical aspects

The study was approved by the Institutional Review Board of the Ramón y Cajal Hospital (Madrid), and conducted in accordance with the principles of the Declaration of Helsinki. All patients signed written informed consent forms to participate in the study.

## Results

Table 1 shows the socio-demographic and clinical characteristics at baseline visit of the MADIABETES CKD Cohort. Females represent 45.2% of the study population. Mean age in this patient cohort was 67.3 years (SD = 10.8) and the average T2DM duration was 8.5 years (SD = 7.4). The vast majority (91.6%) had a low risk (less or equal to 10 percent) of developing coronary events within the next ten years.

**Table 1:** Baseline characteristics of type 2 diabetes mellitus patients included in the cohort.

	**N = 2,620**	**95% CI**
Female gender, (%)	45.2	43.3–47.1
Age (yr), mean (SD)	67.3 (10.8)	66.8–67.7
Duration of DM (yr), mean (SD)	8.5 (7.4)	8.3–8.8
Duration of DM (yr), median (IQR)	6 (7)	6.0–7.0
Current smoker, (%)	18.1	16.6–19.6
Former smoker, (%)	27.4	25.8–29.2
Non-Smoker, (%)	54.5	52.6–56.4
**Medication Profile**, (%)		
Oral antidiabetic	75.2	73.5–76.8
Insulin	17.4	16–19
Antihypertensive agents	81.7	80.2–83.2
Aspirin	48.8	46.8–50.7
Statins	70.8	69–72.6
**History of**, (%)		
Myocardial Infarction	11.2	10–11.4
Stroke	7.1	6.2–8.2
Hypertension	69.7	68–71.5
Congestive heart failure	5.6	4.8–6.6
Neuropathy	6.4	5.5–7.4
Retinopathy	7.7	6.8–8.8
Peripheral arteriopathy	6.2	5.4–7.2
**Risk of developing coronary events at baseline visit**		
Adjusted REGICOR function 10-year risk, mean (SD)	5.9 (2.9)	5.8–6
Proportion patients with risk <5%	52.7	50.6–54.7
Proportion patients with risk 5–10%	38	36–40
Proportion patients with risk >10%	9.4	8.3–10.6
**Anthropometric variables**		
BMI (Kg/m^2^), mean (SD)	30 (5)	29.8–30.2
SBP (mmHg), mean (SD)	133.5 (13.7)	132.9–134
DBP (mmHg), mean (SD)	77.2 (8.1)	76.9–77.6
**Laboratory variables**		
FPG (mg/dl), mean (SD)	144.8 (41.8)	143.1–146.4
FPG (mg/dl), median (IQR)	136 (41)	135–138
Patients with HbA1c level <7, (%)	53.2	51.3–55.1
HbA1c (%), mean (SD)	7.1 (1.2)	7–7.1
HbA1c (%), median (IQR)	6.9 (1.4)	6.8–6.9
Dyslipidemia(%)	57.2	55.3–59.1
Total Cholesterol (mg/dl), mean (SD)	192.8 (35.7)	191.5–194.2
LDL-C (mg/dl), mean (SD)	116 (29.7)	114.9–117.2
HDL-C (mg/dl), mean (SD)	49 (12.6)	48.6–49.5
Triglycerides (mg/dl), mean (SD)	144.3 (94)	140.6–147.8
Triglycerides (mg/dl), median (IQR)	121	118–124
Albuminuria^1^ (%)	20.1	17.4–23.1

^1^Albumin excretion rate >30 mg/g.

SD: Standard Deviation; IQR: Interquartile range; BMI: Body mass index; SBP: Systolic Blood Pressure; DBP: Dyastolic Blood Pressure; FPG: Fasting plasma glucose; HbA1c: Glycated haemoglobin; LDL-C: low-density lipoprotein cholesterol; HDL-C: high-density lipoprotein cholesterol.

Table 2 also shows the characteristics of patients that developed CKD compared with those that did not. There were statistically significant differences between CKD and non-CKD patients with respect to age, duration of T2DM, smoking status, and medication profile (aspirin, antihypertensive agents, and insulin). CKD patients had a higher prevalence of cardiovascular events (myocardial infarction, stroke, peripheral arteriopathy, and congestive heart failure), cardiovascular risk factors (hypertension), and chronic diabetic complications (retinopathy and nephropathy). However, there were no differences between the groups in the use of oral antidiabetic agents, statins, risk of developing coronary events (Score REGICOR function) and control parameters (BP, HbA1c, LDL-cholesterol, BMI). The CKD patients had a significantly higher crude mortality rate (18.3% vs. 8.5%; p<0.001).

**Table 2:** Comparison of characteristics according to the presence of CKD (Stage 3–5).

	**Chronic Kidney Disease** ^1^ **(n = 268)**	**No Chronic Kidney Disease (n = 2,352)**	**p value** *
Female gender, (%)	48.5	44.8	0.247
Age (yr), mean (SD)	71.8 (9.8)	66.7 (10.8)	<0.001
Duration of DM (yr), mean (SD)	10.4 (8.2)	8.3 (7.2)	<0.001
Duration of DM (yr), median (IQR)	8 (9)	6 (7)	<0.001
Current smoker, (%)	11.7	18.8	0.004
Former smoker, (%)	29.7	27.2	0.384
Non-Smoker, (%)	58.6	54	0.152
**Medication Profile**, (%)			
Oral antidiabetic	79.8	74.7	0.068
Insulin	25.2	16.6	0.001
Antihypertensive agents	95.7	80.1	<0.001
Aspirin	55.8	48	0.017
Statins	74.4	70.4	0.183
**History of**, (%)			
Myocardial Infarction	18.7	10.3	<0.001
Stroke	11.6	6.6	0.003
Hipertensión	85.4	68	<0.001
Congestive heart failure	9.7	5.2	0.02
Neuropathy	7.5	6.2	0.44
Retinopathy	12.7	7.2	0.001
Peripheral arteriopathy	11.2	5.7	<0.001
**Risk of developing coronary events**			
Adjusted REGICOR function 10-year risk, mean (SD)	6 (3.4)	5.9 (2.9)	0.504
Proportion patients with risk <5%	53	52.6	0.905
Proportion patients with risk 5–10%	35.1	38.3	0.323
Proportion patients with risk >10%	12	9.1	0.137
**Anthropometric variables**			
BMI (Kg/m^2^), mean (SD)	30.3 (5.3)	30 (4.9)	0.381
SBP (mmHg), mean (SD)	136.7 (13)	133.1 (13.7)	0.288
DBP (mmHg), mean (SD)	76.7 (7.4)	77.3 (8.1)	0.983
**Laboratory variables**			
FPG (mg/dl), mean (SD)	142.5 (45.9)	145 (41.3)	0.381
FPG (mg/dl), median (IQR)	134 (47)	136 (42)	0.288
Patients with HbA1c level <7, (%)	52.3	53.3	0.751
HbA1c (%), mean (SD)	7.1 (1.2)	7.1 (1.8)	0.868
HbA1c (%), median (IQR)	6.9 (1.1)	6.9 (1.4)	0.954
Dyslipidemia, (%)	54.5	57.5	0.336
Total Cholesterol (mg/dl), mean (SD)	191.8 (36.9)	193 (35.5)	0.616
LDL-C (mg/dl), mean (SD)	113.3 (28.8)	116.4 (29.7)	0.12
HDL-C (mg/dl), mean (SD)	49.4 (13)	49 (12.5)	0.619
Triglycerides (mg/dl), mean (SD)	151.8 (100.5)	143.4 (93.2)	0.178
Triglycerides (mg/dl), median (IQR)	126 (79)	120 (77)	0.07
Albuminuria^2^, (%)	36	18	<0.001
Microalbuminuria (%)	30.3	17	0.002
Macroalbuminuria (%)	5.7	1	0.001
**Crude All-Cause Mortality at 5 years (%)**	18.3	8.5	<0.001

^1^. Stage 3–5 of K/DOQI

^2^. Albumin excretion rate > 30 mg/g

SD: Standard Deviation; IQR: Interquartile range; BMI: Body mass index; SBP: Systolic Blood Pressure; DBP: Dyastolic Blood Pressure; FPG: Fasting plasma glucose; HbA1c: Glycated haemoglobin; LDL-C: low-density lipoprotein cholesterol; HDL-C: high-density lipoprotein cholesterol.

* Pearson’s chi-square method was applied for categorical variables, and Student’s t-test for continuous variables. Mann-Whitney U-test was applied to compare medians of duration of DM2.

The cumulative incidence of CKD at five-years was 10.23% (95% CI = 9.12–11.43) and the incidence density was 2.07 (95% CI = 1.83–2.33) cases per 1,000 patient-months or 2.48 (95% CI = 2.19–2.79) cases per 100 patient-years. Among patients living T2DM for <10 years, the cumulative incidence of CKD was 8.48% (95% CI = 7.29–9.84) vs. 14.25% (95% CI = 11.99–16.86) in patients with T2DM for ≥ 10 years. Incidence density was 2.03 cases per 100 patient-years (95% CI = 1.73–2.38) among T2DM <10 years and 3.54 (95% CI = 2.92–4.25) among patients living with T2DM for longer. The cumulative incidence rates stratified by duration of T2DM can be considered significantly different because their 95% CI do not overlap. This was equally the case for incidence density rates.

The adjusted Hazard Ratios (HR) of risk factors associated with incidence of CKD are shown in Table 3. The highest HR was albuminuria ≥ 300 mg/g (HR = 4.57; 95% CI = 2.46–8.48). Furthermore, the other variables with high HR were age over 74 years (HR = 3.20; 95% CI = 2.13–4.81), history of Hypertension (HR = 2.02; 95% CI = 1.42–2.89), Myocardial Infarction (HR = 1.72; 95% CI = 1.25–2.37), dyslipidemia (HR = 1.68; 95% CI = 1.30–2.17), SBP >149 mmHg (HR = 1.52; 95% CI = 1.02–2.24) and duration of T2DM ≥ 10 years (HR = 1.46; 95% CI = 1.14–1.88). Moreover, an exploratory analysis of the metabolic syndrome data showed a non-significant association with incidence of CKD (HR = 1.08, 95% CI = 0.8–1.5; p = 0.617).

**Table 3:** Associated Risk Factors for Incident CKD (Stage 3–5) (n = 268) after five-year follow-up of 2,620 patients (Multivariable Cox Regression).

**Variables**	**aHR**	**HR 95% CI**	**p value**
**Age**			
<60 years	1		
60–74 years	1.86	1.25–2.77	0.002
>74	3.20	2.13–4.81	<0.001
**Albuminuria**			
< 30 mg/g	1		
30–299 mg/g	1.67	1.11–2.50	0.013
≥ 300 mg/g	4.57	2.46–8.48	<0.001
**Gender**			
Male	1		
Female	1.15	0.89–1.49	0.283
**History of prior Hypertension**			
No	1		
Yes	2.02	1.42–2.89	<0.001
**Duration of Diabetes Mellitus**			
<10 years	1		
≥10 years	1.46	1.14–1.88	0.003
**Myocardial infarction**			
No	1		
Yes	1.72	1.25–2.37	0.001
**Dyslipidemia**			
No	1		
Yes	1.68	1.30–2.17	<0.001
**SBP**			
<130 mmHg	1		
130–149 mmHg	1.22	0.91–1.64	0.184
>149 mmHg	1.52	1.02–2.24	0.037

aHR: Adjusted Hazard Ratio; CI: Confidence Interval; CKD: Chronic Kidney Disease; SBP: Systolic Blood Pressure.

Finally, the development of a probability table of CKD risk can be seen in Table 4. The greatest risk is observed in patients over 75 years, with T2DM duration ≥10 years, with hypertension, albuminuria ≥ 300 mg/g, SBP ≥150 mmHg, dyslipidemia and AMI.

**Table 4:** Risk Table: Probability of developing sustained impaired Glomerular Filtration Rate (Stage 3–5 K/DOQI), in five years.

AMI	DISLYPEMIA	BPS	<75 YEARS												≥75 YEARS											
			DURATION OF DIABETES MELLITUS < 10 YEARS						DURATION OF DIABETES MELLITUS ≥10 YEARS						DURATION OF DIABETES MELLITUS < 10 YEARS						DURATION OF DIABETES MELLITUS ≥10 YEARS					
			HYPERTENSION						HYPERTENSION						HYPERTENSION						HYPERTENSION					
			NO			YES			NO			YES			NO			YES			NO			YES		
			ALBUMINURIA			ALBUMINURIA			ALBUMINURIA			ALBUMINURIA			ALBUMINURIA			ALBUMINURIA			ALBUMINURIA			ALBUMINURIA		
			NO	30–299	≥300	NO	30–299	≥300	NO	30–299	≥300	NO	30–299	≥300	NO	30–299	≥300	NO	30–299	≥300	NO	30–299	≥300	NO	30–299	> = 300
NO	NO	< 130	**2.8**	4.4	11.3	5.9	9.3	22.8	4.2	6.7	16.9	8.9	13.9	32.8	5.6	8.8	21.6	11.6	17.9	40.7	8.4	13.2	31.2	17.2	26.2	55.2
		130–149	3.5	5.6	14.2	7.4	11.7	28.0	5.4	8.5	20.9	11.2	17.4	39.6	7.0	11.0	26.6	14.5	22.2	48.6	10.6	16.4	37.8	21.3	32.0	64.0
		> = 150	4.3	6.8	17.1	9.0	14.1	33.1	6.5	10.3	25.0	13.5	20.8	46.1	8.5	13.3	31.5	17.4	26.5	55.7	12.8	19.8	44.1	25.5	37.7	71.4
	YES	< 130	4.4	7.0	17.4	9.2	14.4	33.7	6.7	10.5	25.5	13.8	21.2	46.8	8.7	13.6	32.1	17.8	27.0	56.5	13.0	20.1	44.8	26.0	38.3	72.2
		130–149	5.6	8.8	21.6	11.6	17.9	40.7	8.4	13.2	31.2	17.2	26.2	55.2	10.9	17.0	38.9	22.0	33.0	65.3	16.3	24.9	53.1	31.8	45.9	80.3
		> = 150	6.8	10.6	25.8	14.0	21.5	47.3	10.2	15.9	36.7	20.6	31.1	62.6	13.2	20.4	45.3	26.3	38.7	72.7	19.6	29.6	60.4	37.4	52.9	86.4
YES	NO	< 130	4.7	7.4	18.5	9.8	15.3	35.6	7.1	11.2	27.0	14.7	22.6	49.2	9.3	14.5	33.9	18.9	28.6	59.0	13.9	21.4	47.1	27.5	40.4	74.6
		130–149	5.9	9.4	22.9	12.3	19.1	42.9	9.0	14.0	33.0	18.3	27.8	57.7	11.7	18.1	41.0	23.4	34.8	67.8	17.3	26.4	55.5	33.6	48.2	82.5
		> = 150	7.2	11.4	27.3	14.9	22.8	49.6	10.9	16.9	38.7	21.9	32.9	65.1	14.1	21.7	47.6	27.8	40.8	75.1	20.8	31.3	62.9	39.4	55.4	88.2
	YES	< 130	7.4	11.6	27.8	15.2	23.3	50.4	11.1	17.3	39.4	22.4	33.4	65.9	14.4	22.1	48.3	28.4	41.5	75.8	21.2	31.9	63.7	40.1	56.2	88.7
		130–149	9.3	14.5	33.9	18.9	28.6	59.0	13.9	21.4	47.1	27.5	40.4	74.6	17.9	27.2	56.8	34.6	49.4	83.5	26.1	38.6	72.5	47.9	64.9	93.7
		> = 150	11.2	17.5	39.8	22.6	33.8	66.4	16.8	25.5	54.2	32.6	47.0	81.3	21.5	32.2	64.2	40.5	56.6	89.0	31.0	45.0	79.4	55.0	72.3	**96.7**

**AMI**: Acute Myocardial Infarction

**SBP**: Systolic Blood Pressure

Bold type indicated the minimum and maximum values.

The Harrell’s C-index value for the CKD model was 70.3 (95% CI = 66.5–73.9), this implies a good prediction ability to discriminate between patients with and without CKD.

## Discussion

### Incidence of CKD

Our CKD (stage 3–5) incidence density is high (2.48 cases per 100 patient-years) compared to other studies among T2DM patients that reported a lower incidence density, between 0.133 to 0.200 cases per 100 patient-years [19,20]. These differences may be explained, in part, as these studies use data from the 1990s when the prevalence of impaired renal function in diabetic patients was lower than today [21]. The higher current prevalence of reduced GFR is because the age of onset of type 2 diabetes is decreasing, allowing patients enough duration of diabetes to develop microvascular complications. Furthermore, survival of diabetic patients with overt diabetic nephropathy has been improved therefore increasing the prevalence [22]. Also, recently, de Boer et al. has shown incidence densities between 0.16 per 100 patient-years in subjects from the DCCT/EDIC study assigned to intensive therapy and 0.3 per 100 patient-years in those assigned to conventional therapy [23].

The cumulative incidence rate at 5 years was 10.23% (2.04% / year) in our study. This rate is concordant with rates found in previously published literature among patients with T2DM. For example, in a Swedish observational population-based study, Afghahi et al. found that 11% (2.2% / year) of 3,667 T2DM patients developed renal impairment [24]. Also, a recent analysis of 1,449 patients with T2DM performed within the framework of the Verona Diabetes Study [25] reported a 13.4% incidence of developed CKD during a mean follow-up of 5 years. In the UKPDS-74, 29% developed renal impairment (1.93%/year) after a median follow-up of 15 years [26].

Patients with long duration of T2DM showed higher cumulative incidence and incidence density of CKD than patients who were recently diagnosed. These findings are in agreement with published studies [5], showing that patients diagnosed with T2DM for more than 15 years show an annual decline of eGFR of -1.0 mL/min per 1.73 m^2^ per year and a -1.4 annual eGFR decline (% per year) compared to -0.7 mL/min per 1.73 m^2^ per year and -0.8 annual eGFR decline (% per year) in more recently diagnosed patients. However, other studies did not find an association between duration of T2DM and eGFR decline [27].

### Risk Factors

In the present Cohort, the main variables associated with incidence of CKD stage 3–5 were albuminuria ≥ 300 mg/g and age >74 years. Several predictive models of kidney failure have included age and history of albuminuria, both in the general population and in T2DM patients [24,26–28]. However, Tangri et al. [16] identified age as a protective factor for developing kidney failure, defined as the need for dialysis or kidney transplantation. In this study the HR was 0.61 (p<0.05) for each increase of 5 mL/min/1.73m^2^. This finding is not consistent with the vast majority of studies, and is probably due to the fact that dialysis and transplantation are primarily offered to younger patients.

Albuminuria (microalbuminuria and macroalbuminuria) is a well-established risk factor for deterioration of renal function. Observational studies have shown a relationship between increasing albuminuria and declining GFR. In T2DM patients, a relative risk of 3.6 (95% CI = 1.6–8.4) has been identified [28]. In addition, a greater decline in GFR has been seen in patients with microalbuminuria (1.7 ml per minute per year) compared with those that have normoalbuminuria [28].

Similarly to previous studies, our data show a comparable risk value of albuminuria ≥ 300 mg/g for developing CKD (HR = 4.57; 95% CI = 2.46–8.48). On the other hand, dissociation between albumin excretion rate and GFR has been reported in T2DM patients [29,30].

As is documented in the specialist literature [31], albuminuria > 300 mg/g is a well established risk factor for CKD incidence and this is reflected in our data. Thus, we strongly advise health care providers to increase their efforts to prevent and/or reverse albuminuria in their patients. In this line, a pooled analysis of interventional studies has demonstrated that the initial percentage decrease in albumin excretion rate was 20.8% in T2DM with late diabetic nephropathy (macroalbuminuria) and the corresponding annual percentage rate of decline in GFR was 9.2% [32].

Additionally, two studies demonstrated that some patients with DM develop a low creatinine clearance while remaining normoalbuminuric [33,34]. Similarly, in the UKPDS-74 Study the majority (51%) of patients who developed CKD stage 3–5 did not have preceding microalbuminuria [26].

Hypertension is both a cause and effect of renal impairment. A meta-analysis of several large intervention studies has shown that a lower mean BP during therapy is associated with slower rates of decline of GFR [35]. One standard deviation increase in 24h ambulatory SBP increases the risk of end- stage renal disease by 3.04 (95% CI = 2.13–4.35) and by 2.20 (95% CI = 1.43–2.39) when it is adjusted for standard clinic SBP [36]. Also, BP control has been shown to reduce the incidence of albuminuria and for each decrement of 10 mmHg of SBP the incidence of CKD is reduced by approximately 10% [37]. Numerous studies have reported an increased risk of CKD in hypertensive patients [24,38–40] with the HR ranging from 1.4 to 3.0. Our study showed that having SBP in the upper range (>149 mmHg) was associated with an approximately 50% higher CKD incidence (HR = 1.52; 95% CI = 1.02–2.24; p = 0.037). Herget-Rosenthal et al. [41] found hypertension to be a risk factor (HR 4.83; CI 95% = 1.18–19.35) to developing eGFR decline >7.5 mL/min/1.73 m^2^ in high risk primary care patients after three years follow-up. However, other studies did not find a relationship between SBP and incidences of CKD 3–5 after adjustment for age [42].

Dyslipidemia has exhibited inconsistent association with CKD stage 3–5 incidence in the general population and diabetic cohorts. While a large body of evidence suggests that dyslipidemia has an important role in the progression of kidney disease in patients with diabetes [43], other studies have shown that high triglycerides [44] and low HDL cholesterol, but not LDL cholesterol, predict an increased risk of renal dysfunction [45] and decline of GFR [46]. In fact, the largest study evaluating this question included 2,193 patients with T2DM and normal renal function at baseline. In this study, each 0.26 mmol/L higher level of HDL was associated with a 24% lower risk of developing stage 3 CKD [47]. In our cohort, dyslipidemia increased the risk of CKD by 68% (HR = 1.68; CI 95% = 1.30–2.17; p<0.001). However, the UKPDS 74 study did not find this association [26].

Female gender is usually significantly associated with a decline of GFR [24,26,40]. However, other studies have found a higher association with male gender [42]. We found a non-significant association between female gender and Stage 3–5 CKD incidence (HR = 1.15; CI 95% = 0.89–1.49; p = 0.283).

Coronary Heart Disease has been shown to be a risk factor to developing CKD [48]. The time course of renal function after Myocardial Infarction, adjusted for baseline GFR, has been well defined: a loss of renal function of 2–3 ml min^−1^ year^−1^ in the first days after an acute MI, with a subsequent steady decline of approximately 3 ml min^−1^ year^−1^ in excess of the normal age-related rate of renal function loss [49]. However, several studies have not found this association [16,24,40–42,50,51]. In the present study, myocardial infarction showed a HR of 1.72 (95% CI = 1.25–2.37; p<0.001).

The association between CKD and Acute Myocardial Infarction incidence is well established with HRs ranging from 1.4 (GFR 45–59 mL/ min/ 1.73 m^2^) to 3.4 (GFR <15 mL/ min/ 1.73 m^2^) [52].

The effect of CKD (stage 3–5) on mortality rate at 5 years (18.3% vs. 8.5%; p<0.001) is consistent with previous reports [53–56] that show a more than 2-fold increase in mortality rate in patients with reduced GFR compared to patients with normal GFR.

Although a recent meta-analysis [57] showed that Metabolic Syndrome was associated with development of an eGFR <60 mL/ min/ 1.73 m^2^, with OR = 1.55 (95%CI = 1.34–1.80), our study did not find such an association. Thus, metabolic syndrome was not included in our model and we instead decided to include the history of hypertension and dyslipidemia.

Finally, other factors such as the Mediterranean diet may be considered a factor in the development of CKD incidence in patients with T2DM. For example, Dunkler et al. [58] show, using the modified Alternate Healthy Eating Index (mAHEI) tool, that compared to participants in the least healthy mAHEI score tertile, participants in the healthiest tertile had a lower risk of CKD (OR = 0.74; 95% CI = 0.64–0.84) and a lower risk of mortality (OR = 0.61; 95% CI = 0.48–0.78). Also, participants consuming more than 3 servings of fruit per week had a lower risk of CKD compared with participants consuming these food items less frequently. In this sense it is possible that the Mediterranean diet had a similar effect. However, different studies in Mediterranean populations have not shown lower incidence rates of CKD compared to non-Mediterranean populations [59]. In our study, we did not collect data on food consumption, so we cannot analyze the effect of diet.

### Risk Table

To our knowledge, this is the first study that has developed a simple risk table to predict five-year CKD risk in patients with T2DM. Taking a high-risk person as an example: age over 75 years old, hypertension, myocardial infarction, albuminuria ≥ 300 mg/g, dyslipidemia, duration of T2DM ≥ ten years, and SBP ≥150 mmHg; the risk of developing CKD at five-year follow-up is 96.7%. However, in the same patient, without albuminuria the risk is reduced to 55%, and without albuminuria and SBP <130 mmHg the risk is further reduced to 40.1%. Thus, after changing SBP and albuminuria values the risk decreases from 96.7% to 40.1% (56.6 percentage points less).

This risk table enables the calculation of individual risk estimates and can help in the development of risk reduction strategies by highlighting certain parameters such as SBP, or albuminuria regression, that need controlling. Therefore, it is particularly useful for general practitioners caring for patients with T2DM in our setting, as it allows the monitoring of risk over time and could be used by general physician at primary health care centers to identify high risk patients and improve prevention of CKD.

### Strengths and Limitations

There are several potential implications of this paper. Selection bias is an important limitation to this study and may affect the external validity of our results. The study sample was not population based as it was drawn from outpatients visiting Primary Health Care Centers. Patients who attend private clinics, who may have a higher socioeconomic status, are possibly under-represented in our sample. Furthermore, the study was conducted in Madrid, a large city in Spain, which may not represent the entire Spanish population in terms of income, educational level, lifestyle, and health profile.

The MADIABETES cohort is well described, made up of people living with T2DM and analyzed under usual clinical practice conditions. As expected, some patients had incomplete serum creatinine data at baseline (n = 36; 1%) or follow-up (n = 49; 1.4%). Owing to this feature of the study, patients were observed until last contact with general practitioner or until death; a total of 249 subjects died during follow-up. This must be considered when generalizing the study findings.

Finally, it is important to consider that some confounding variables, such as compliance with medical treatments, may not have been collected in this study and we cannot completely rule out the possibility of residual confounding. Also, it is necessary to validate the multivariate model in a simple over a longer time frame in order to improve accuracy and reliability.

Importantly, general practitioners can improve the quality of care delivered to their T2DM patients by being able to calculate risk probabilities. This will enable them to provide better clinical counseling and improve decision-making. Strengths of this study include the prospective design, which ensured that measurement of risk factors preceded the development of impaired eGFR. In addition, the requirement of sustained impaired eGFR (one event of eGFR <60 mL/ min/ 1.73 m^2^ and average of successive eGFR less 60 mL/ min/ 1.73 m^2^) when defining outcomes helped improve the specificity, because a decrease in eGFR may represent transient changes in kidney perfusion or function that are not necessarily related to the casual pathway that is driving the development of diabetic kidney disease.

## Conclusions

After a five-year follow-up, the cumulative incidence of CKD is concordant with rates previously published in the literature. Age over 74 years and albuminuria ≥ 300 mg/g were the risk factors more strongly associated with incidence of CKD stage 3–5. Our results suggest that a greater control of BP, lipid and albuminuria could reduce the incidence of CKD in patients with T2DM. Further studies are necessary to confirm the role of these variables as risk factors and elucidate their causal relationship on CKD.
